# Parasomnias and Disruptive Sleep-Related Disorders: Insights from Local Sleep Findings

**DOI:** 10.3390/jcm11154435

**Published:** 2022-07-29

**Authors:** Serena Scarpelli, Valentina Alfonsi, Maurizio Gorgoni

**Affiliations:** 1 Department of Psychology, Sapienza University of Rome, Via dei Marsi 78, 00185 Rome, Italy; valentina.alfonsi@uniroma1.it (V.A.); maurizio.gorgoni@uniroma1.it (M.G.); 2 Body and Action Lab, IRCCS Fondazione Santa Lucia, 00179 Rome, Italy

Parasomnias are sleep disorders that involve abnormal behaviors, emotional experiences, perceptions, and dream activity, occurring during specific sleep stages or sleep–wake transitions. The third edition of the *International Classification of Sleep Disorders* (ICSD-III, 3rd ed.) [1] categorizes parasomnias into three clusters: non-rapid eye movement (NREM) related, rapid-eye movement (REM) related, and others (i.e., sleep state-independent).

Recent research introducing the concept of local sleep regulation [2] has offered new perspectives in the research field of parasomnia and parasomnia-like phenomena. Indeed, human sleep does not necessarily represent a global process since wake-like and sleep-like local electrophysiological features can co-exist in different cerebral regions during sleep, wakefulness, and transitions between these states [3,4,5]. It has been recently proposed that several NREM sleep parasomnias, such as sleepwalking, sleep terrors, and confusional arousals, recognized as disorders of arousal (DOAs), may represent a privileged context to study local dissociated electrophysiological patterns and their relationships with the modulation of vigilance and behaviors [6]. A limited number of polysomnographic (PSG) studies found an increase in sleep fragmentation and an atypical electroencephalographic (EEG) slow wave activity (SWA) in individuals with NREM parasomnias compared to healthy subjects [7]. At present, the knowledge about the neural correlates of NREM parasomnias is still inconclusive. However, the research interest in the local electrophysiological features of NREM parasomnias is growing [7,8,9], and we believe that a further effort in the investigation of regional EEG patterns before parasomnia episodes could provide new insights into the functional significance of NREM parasomnias.

Concerning REM sleep parasomnias, local sleep may represent an interesting perspective on REM sleep behavior disorder (RBD). This parasomnia is characterized by vivid dream activity and dream enactment provoked by the loss of physiological REM atonia (ICSD-III, 3rd ed.) [1], and it can be classified as isolated (iRBD) when it cannot be ascribed to any other condition [10]. Notably, the iRBD represents a prodromal manifestation of neurodegenerative diseases that fall under alpha-synucleinopathies, such as Parkinson’s disease and Lewy body dementia [11]. Therefore, a clinically relevant research question concerns the identification of possible biomarkers able to predict neurodegeneration in iRBD patients. Among the EEG findings, the most frequently reported result is characterized by a wide EEG that slows during wakefulness and REM sleep and is associated with cognitive functioning. This is helpful in identifying patients with a higher risk of progression in alpha-synucleinopathies [12]. Evidence about NREM sleep peculiarities in iRBD is conflicting [12]. Nevertheless, the recent application of a topographical approach allowed the identification of regional EEG alterations during NREM sleep in iRBD [13,14,15]. Starting from the growing literature about the role of local NREM sleep oscillations in neurodegenerative disorders such as Alzheimer’s Disease [16] and their possible implications in rehabilitation-dependent plasticity and sleep-based therapeutic strategies [17,18], these findings suggest that an assessment of the local sleep pattern may represent a promising strategy to increase our knowledge of this REM parasomnia, its relationship with the development of neurodegenerative disorders, and its possible role in novel therapeutic approaches.

Another potential application of the assessment of local sleep in parasomnias concerns the field of dream research. By definition, dream content is not directly accessible. Therefore, researchers obtain information on oneiric activity by retrospective recall: spontaneous or provoked awakenings are the gold standard for dreaming investigation [19]. However, the retrospective collection of dream reports brings some biases, such as distortions or omissions due to memory reprocessing [20], and it is difficult to determine when a recalled dream was produced during a night of sleep. The literature regarding parasomnias has paved the way for a new method of investigating mental activity during sleep. The behavioral activations of parasomnias are sometimes connected to specific dream features (i.e., dream-enactment behaviors (DEBs)) [21]. Thus, parasomnias could give us a unique opportunity to directly assess ongoing mental activity during sleep [22]. Since the regional sleep EEG pattern can predict the following dream recall [23,24,25,26,27,28], in our opinion, the assessment of local electrophysiological features of parasomnia events associated with dream recall may represent a new frontier in dream research, potentially providing new insight on the neurobiology of dreaming and its relationship with daily experience.

Overall, local sleep represents an interesting perspective to increase our understanding of parasomnias. Beyond such a psychophysiological view, the complexity of these phenomena, their frequent underdiagnosis, and poor understanding (particularly concerning DOAs) lead to the need for further efforts and the integration of different clinical and scientific perspectives to promote new knowledge in this field.

The present Special Issue aims to provide updates on different facets of parasomnia and parasomnia-like events. Original articles and reviews focused on local electrophysiological patterns of parasomnias are encouraged. Moreover, the submission of articles focused on different aspects of parasomnias and disruptive sleep-related disorders is welcome, including: brain mechanisms; pathogenesis; nocturnal and diurnal behavioral, emotional, and physiological features; cognitive functioning; dreaming; neuropsychological patterns; relationships with psychiatric disorders; and pharmacological and non-pharmacological treatments.
